# Intrinsic Control of Surface Immune and Epithelial Homeostasis by Tissue-Resident Gut Stromal Cells

**DOI:** 10.3389/fimmu.2019.01281

**Published:** 2019-06-19

**Authors:** Yosuke Kurashima, Daisuke Tokuhara, Mariko Kamioka, Yutaka Inagaki, Hiroshi Kiyono

**Affiliations:** ^1^Department of Innovative Medicine and Department of Immunology, Graduate School of Medicine, Chiba University, Chiba, Japan; ^2^International Research and Development Center for Mucosal Vaccines, The Institute of Medical Science, The University of Tokyo, Tokyo, Japan; ^3^Department of Mucosal Immunology, IMSUT Distinguished Professor Unit, The Institute of Medical Science, The University of Tokyo, Tokyo, Japan; ^4^Division of Gastroenterology, Department of Medicine, CU-UCSD Center for Mucosal Immunology, Allergy and Vaccines (cMAV), University of California, San Diego, San Diego, CA, United States; ^5^Laboratory of Vaccine Materials and Laboratory of Gut Environmental System National Institutes of Biomedical Innovation, Health and Nutrition, Osaka, Japan; ^6^Department of Pediatrics, Osaka City University, Graduate School of Medicine, Osaka, Japan; ^7^Center for Matrix Biology and Medicine, Graduate School of Medicine, Tokai University, Isehara, Japan

**Keywords:** mucosal immunology, mesenchymal cells, fibroblasts, intestinal stem cells (ISCs), Peyer's patches

## Abstract

The epithelial layer creates a chemical and physical barrier at the forefront of intestinal mucosa, and immune cells beneath the surface epithelium are poised to react to extrinsic factors, to maintain tissue homeostasis. Importantly, the nexus of epithelial–immune responses at mucosal surfaces is dexterously modulated by intrinsic stromal–mesenchymal cells. First, organogenesis of lymphoid tissues, including Peyer's patches, requires dynamic interplay between lymphoid cells and stromal cells, which have become known as “lymphoid organizers.” Second, correct spatiotemporal interaction between these cell populations is essential to generate the infrastructure for gut immune responses. Moreover, immune cells at the intestinal barrier are functionally modulated by stromal cells; one such example is the stromal cell–mediated differentiation of innate immune cells, including innate lymphoid cells and mast cells. Ultimately, mucosal stromal cells orchestrate the destinations of epithelial and immune cells to maintain intestinal immune homeostasis.

## Introduction

The single layer of epithelial cells at the intestinal mucosa creates both a chemical and physical barrier to protect the body. Overlying the epithelial surface of the digestive tract are mucin-containing layers, which play an important role in preventing commensal bacteria from attaching directly to gut epithelium. A single layer of mucus covers the small intestinal epithelium, whereas two layers (inner and outer) overlay the epithelium of the stomach and colon (1). Mucus is net-like in structure and forms a gel due to its glycoprotein components, MUC2 (secreted by goblet cells in the intestine and colon) and MUC5AC (produced by gastric epithelial glands in the stomach) (2, 3). In addition, the mucus layer in the intestinal compartment contains anti-microbial peptides, including α-defensin, which is produced by Paneth cells in the intestinal crypts located at the base of villi (4), and secretory IgA molecules, which derive from the intestinal mucosa or bile duct (5, 6). A lack of either of these secretory components leads to spontaneous intestinal inflammation or colitis, with insufficient segregation of pathologic and commensal bacteria from the intestinal epithelial layer (7, 8). Therefore, the chemical barriers mounted by mucus layers—the components of which originate from epithelial cells and are mediated by secretory systems—are required for both the maintenance of intestinal homeostasis and protection from infection.

**Figure 1:**
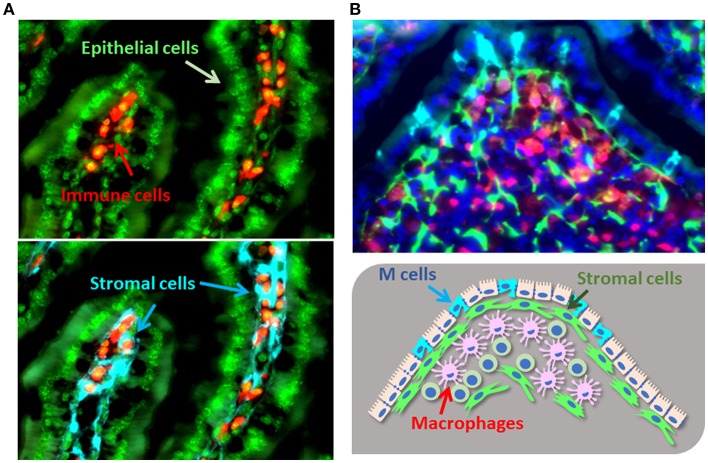
Cellular localization of gut stromal cells. **(A)**. The colocalization of gut immune cells [e.g., B cells (CD19), red] and epithelial cells (DAPI, green) with stromal cells (type I collagen, light blue) in murine intestinal villi. **(B)**. Immune cells [e.g., macrophages (lysozyme), red] and stromal cells (type I collagen, green) reside under the M cells located in the FAE of PPs (GP2, light blue; DAPI, dark blue).

To efficiently absorb nutrients from the ingested diet, the intestine contains a huge number of villi. The gut epithelial cells overlying these villi create a physical barrier by sealing the spaces together with other epithelial cells [reviewed in (9)]. These seals are the so-called cellular junctions, comprising both adherent and tight junctions. Various molecules within cellular junctions, such as E-cadherin, regulate epithelial permeability, such that disruption or genetic polymorphism of these molecules increases the susceptibility to intestinal inflammation (10). Stromal cell populations are located beneath these epithelial cells and the lamina propria (Figure 1A). One key stromal cell population called ISEMFs (intestinal sub-epithelial myofibroblasts) is located subjacent to the basement membranes of the intestinal epithelial cells (Figure 1A) (11). ISEMFs express Acta2 (α-smooth muscle actin); are phenotypically similar to smooth muscle; initiate villous contractions; express various receptors for cytokines, including TNFα and IL-1β; and sense epithelial barrier damage and inflammation (11, 12). Consequently, ISEMFs are involved in several steps of wound healing, including epithelial restitution and epithelial stem-cell proliferation (13, 14). During the process of epithelial restitution, epithelial cells from intestinal crypts rapidly migrate to cover and seal epithelium-denuded areas of intestine in the absence of epithelial proliferation [reviewed in (15, 16)]. In addition, ISEMF-like stromal cells are located beneath the basal membrane of the epithelial layer covering Peyer's patches (PPs), which is known as FAE (follicular associated epithelium) (Figure 1B). FAE includes a unique cell population harboring short microvilli, named microfold (M) cells [reviewed in (17, 18)]. These short microvilli are readily accessible to luminal bacteria, which express ligand molecules (e.g., on fimbriae) for attaching to the apical surface of M cells through specific receptors (e.g., glycoprotein 2 [GP2]). Stromal cells are involved in the development and maintenance of M cells and in intestinal mucosal immune responses (19). Through these functions, stromal cells act not only as a second barrier at the intestinal surface but also as determinants of the destinations of diverse immune cells to maintain immune homeostasis (20).

In this review, we highlight current knowledge regarding the crucial and complex roles of stromal cells from intestinal epithelial homeostasis to surface immune responses.

## Spatiotemporal Mucosal Regulation by Intestinal Stromal Cells

As indicated earlier, the stromal cells called ISEMFs are located beneath intestinal epithelial cells (11). ISEMFs express vimentin and Acta2 and contract after epithelial damage to limit the exposed wound area [reviewed in (12, 20)]. ISEMFs produce several growth factors, TGF-β1 and amphiregulin, and support epithelial proliferation by sensing cytokines associated with intestinal damage (e.g., TNFα and IL-1β) (21). In fact, *in vitro* co-culture of ISEMFs and epithelial cells or intestinal organoids (i.e., mini-gut) composed of epithelial cells shows that ISEMFs are critical for epithelial proliferation (13, 22). Furthermore, ISEMFs support the morphology of epithelial cells and the intestinal epithelial lining, because they produce and deposit various types of collagen, including types I, III, IV, V, and VI (23). Collagen types I and III are ubiquitous interstitial collagens and enhance epithelial cell growth (23), whereas type IV contributes to the formation of epithelial basement membranes, and type V is a pericellular collagen for thickening of the intestine wall (24). In addition, loss of collagen VI alters epithelial cell morphology (25). These cytokine-mediated biologic effects on and collagen-mediated physical support of epithelial cells by ISEMFs lead us to consider ISEMFs as a secondary barrier that harmoniously interacts with and promotes the epithelial cell defense function in the mucosal surface.

Stromal cell function is precisely regulated by the local tissue environment. In fact, the genes expressed differ among stromal cells according to their tissue location (26, 27). This remarkable difference in gene expression is particularly evident when comparing stem cell–related molecules (26). Expression levels of cytokines responsible for maintaining intestinal stem cell niches—that is, those involved in Wnt signaling (e.g., WNTs 2b and 5a and WNT agonists [e.g., R-spondins 1 and 3]) and BMP (bone morphogenetic protein) antagonists (e.g., Noggin, Gremlins [GREM] 1 and 2)—differ significantly among various villous regions (e.g., from tip to crypt) (26). Gene analysis of dissected human colonic tips and crypt compartments reveals that genes highly expressed in tips typically are induced by interruption of Wnt signaling through genes induced by dominant-negative transcription factor (TCF) 4 (e.g., p21, a gene that inhibits cell proliferation) and BMP2 (26). Furthermore, genes highly expressed in colonic crypts usually are repressed by dominant-negative TCF4 (e.g., MYC and Cell Division Cycle Associated 7, two genes involved in cell-cycle regulation) and the BMP antagonists GREM1 and GREM2 (26). Therefore, in small intestine, Paneth cells primarily and mesenchymal cells secondarily secrete niche factors (e.g., EGF, WNT3, and the Notch ligand Dll4); in contrast, mesenchymal cells are predominantly responsible for maintaining the stem cell niche in colon, which is devoid of Paneth cells (28, 29). These findings demonstrate the spatiotemporal regulatory mechanisms of stromal cells in creating intestinal stem cell niches.

Directly underneath LGR5-expressing intestinal stem cells lie myofibroblasts and pericryptal stromal populations, which lack Acta2 expression but express CD34, podoplanin, and PDGF (platelet-derived growth factor) receptor α, and the WNT agonist R-spondin 3 (30). These cell populations also produce the winged-helix transcription factor named Foxl1 (forkhead box l1) (30), and a deficiency of Foxl1-expressing stromal cell populations leads to reduced production of niche factors (e.g., R-spondin 3, GREM1, WNT2b, WNT5a) in the crypt compartment. Importantly, Foxl1-deficient mice showed aberrant crypt structure, with ectopic and increased expression of Ephrin-B2 and Ephrin-B3 in epithelial cells (31). These factors are important for epithelial cell positioning along the crypt–villus axis, and their deficiency leads to intermingling of the proliferative and differentiated epithelial cell populations (32). These findings indicate various components of the spatiotemporal regulatory mechanism for stromal cells that ensures adequate stem cell niches and the maintenance of epithelial organization and integrity.

Recently identified additions to the stromal cell populations surrounding intestinal crypts are Foxl1-expressing telocytes (33). Telocytes are a unique type of interstitial cells, which also are found in reproductive tissues, including uterus and placenta [reviewed in (34, 35)]. Telocytes are characterized as having several very thin and long projections, called telopodes. In addition, like other stromal cells, telocytes express CD34, PDGF receptor α, and (typically) c-kit; however, gut telocytes do not express c-kit, unlike the interstitial cells of Cajal (36). Recent evidence reveals the importance of telocytes as a key producer of Wnt ligands in the intestinal crypt (33). Conditional deletion of porcupine, which shows homology to a family of o-acyl transferases that are involved in lipid modification and are required for Wnt production, from Foxl1^+^ cell populations, including telocytes, abolishes the proliferation of stem and transit amplifying cells (33). Indeed, telocytes are absent during the active stage of Crohn's disease, and this lack is correlated with subsequent architectural disruption (37). In addition to those cellular populations, GLI family zinc finger 1 (Gli1) – and Acta2-expressing stromal cells similar to myofibroblasts are involved in maintaining stem cell niches through their production of WNT2b (38, 39). Disruption of Wnt in stromal populations that express Gli1 ameliorates colonic stem cell renewal as well as colonic epithelium integrity (39).

Recently, precise anatomical and immunohistochemical analyses performed in rat intestinal tissue have confirmed the presence of CD34^+^Acta2^−^ populations at the crypt base, PDGFR^high^Acta2^+^ cells laterally along the crypt, and PDGFR^high^Acta2^−^ populations at the tips of villi (40). Due to targeting of porcupine, aberrant secretion of Wnt from PDGFR-expressing pericryptal myofibroblasts ameliorates crypt formation in the neonatal mouse gut (41). However, this experimental system does not exclude the important contributions of other stromal cell populations (e.g., telocytes), thus implying that multiple stromal subsets are involved in the complex and compensative machinery for maintaining both the stem cell niche in the crypt region and epithelial integrity (Figure 1).

In addition to cell-targeting experiments, recent single-cell analysis has revealed precise stromal cell populations in the colons of humans and mice (27). In addition to myofibroblasts, at least 4 newly identified subsets of fibroblasts present in both mice and human colon have been characterized: SOX6^−^CCL8^+^FABP5^high^; BMP2^high^WNT5A^high^; BCL2^high^ OGN^high^; and CD74^high^CCL19^high^ (27). SOX6^−^CCL8^+^FABP5^high^ fibroblasts produce elastic fibers and fibrillar collagen and distribute throughout lamina propria, indicating the involvement of this population in the maintenance of structural and mechanical properties of colon tissue (27). Among those newly identified fibroblasts, BMP2^high^WNT5A^high^ fibroblasts express collagen for epithelial basement membrane (e.g. COL4A5 and COL4A6), predicted as its involvement in epithelial barrier formation (27). Indeed, those populations located near intestinal epithelium. Importantly, this population reduces in the UC patients, implicating that reduction of WNT5a production worsen crypt reconstitution and epithelial integrity (26).

On the contrary, CD74^high^CCL19^high^ fibroblasts expand in the UC patients (27). This population upregulates expression of TNF superfamily member 14 (LIGHT) and release IL-6 and IL-33 during colonic inflammation (27). IL-6 and LIGHT stimulation to the intestinal organoid under the condition mimicking inflammation (lacking Wnt) results in the reduction of epithelial proliferation and upregulation of stem cell maker gene expressions (e.g., LGR5) in organoid. Those results imply that the activation of CD74^high^CCL19^high^ fibroblasts lead to the production of quiescent label-retaining cells, precursor of secretory-type enterocytes (e.g., goblet cells and Paneth cells) (27).

**Figure 2:**
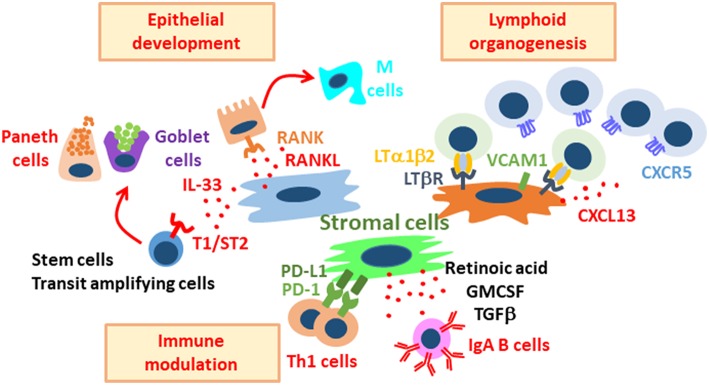
Diverse roles of stromal cells in the regulation of intestinal immunity and physiology. Stromal cells support intestinal epithelial development, lymphoid organogenesis, and immune modulation. These stromal cell populations are distinct, and each population plays important roles in the maintenance of gut homeostasis through their interaction with stem, epithelial, and immune cells.

Furthermore, IL-33 production from fibroblasts is mediated by IL-1β, IL-6, TNF-α, and bacterial cell components (e.g., lipopolysaccharide) (42). IL-33, in turn, stimulates ST2-expressing cryptal epithelial cells and the subsequent development of secretory-type enterocytes, including Paneth cells and goblet cells (42) (Figure 2). Collectively, during inflammation and pathogenic infection, the production of both anti-microbial peptides and mucus is enhanced by fibroblasts that sense “danger signals” (e.g., IL-33). In addition, Paneth cells in the small intestine and a c-kit^+^ subset of colonic goblet cells support intestinal epithelial stem cells via secretion of Wnt, indicating that activation of cryptal stromal cells due to danger signaling leads to both protection and maintenance of the stem cell niche through dual pathways (directly and indirectly through secretory cell–derived Wnt) (43).

However, increase of IL-6 in colonic tissues, produced by continuously activated CD74^high^CCL19^high^ fibroblasts, may strongly influence on the function of macrophages and Th17 cells leading chronic inflammation. So that, regulation of the function of this fibroblast population will be useful therapy for IBD.

Stromal cells located the sub-epithelial compartments of PPs are required for the development of specific epithelial cells, including antigen-sampling M cells. M cells are considered to be “gatekeepers” of the mucosal immune system and serve as entry sites for luminal antigens and bacteria (Figure 1B). M cells have short microvilli and lack the ability to produce anti-microbial peptides and mucus, thus facilitating the attachment of bacteria to their apical surfaces (18). Indeed, M cells express various receptors and guidance molecules for both commensal and pathogenic bacteria (9). For example, GP2 is preferentially expressed on M cells and interacts with the fimbriae of bacteria (44, 45); antigen-presenting cells (e.g., dendritic cells) beneath M cells further capture and process bacterial antigens for the initiation of antigen-specific immune responses in PPs. Therefore, GP2-mediated translocation of bacteria leads to efficient induction of antigen-specific mucosal immune responses (e.g., IgA) (45). The receptor activator of NF-kB (RANK)–RANKL pathway is critically important for the development and differentiation of M cells (46). In particular, stimulation of gut organoids with RANKL revealed the essential roles of the RANK–RANKL pathway for the development of M cells from intestinal epithelial stem cells (47). Whereas, RANK is expressed on all intestinal epithelial cells, RANKL-expressing cells accumulate in the FAE region, and mice lacking either of those molecules showed decreased numbers of M cells in the FAE of PPs (46). Furthermore, RANKL deficiency in type VI collagen–expressing (Col6a1–Cre) cell populations, including M cell–inducing populations, led to the disappearance of M cells (19) (Figure 2). Importantly, those gene-modified mice showed decreased luminal IgA against commensal bacteria (19); therefore, M cell development, as orchestrated through stromal signaling, is a plausible initial pathway through which PPs become able to mediate acquired mucosal immunity.

Overall, current knowledge supports that—as determinants of epithelial integrity and sentinels of mucosal immune responses—sub-epithelial and cryptal stromal cells play indispensable roles in the barrier function of the intestinal mucosa.

## Stromal Network for Mucosal Lymphoid Organogenesis

In addition to epithelial regulation, another important role of stromal cells is their biologic contribution to lymphoid organogenesis [reviewed in (48)]. The organization of lymphoid structures is precisely programmed through molecular and cellular components that are achieved through a stromal network that orchestrates lymphocyte entry into and migration within tissues (49); reviewed extensively in Chang and Turley (50), van de Pavert and Mebius (51), Roozendaal and Mebius (52).

Lymphoid tissue contains 3 types of stromal cells: fibroblastic reticular cells, follicular dendritic cells, and marginal reticular cells (52). Analysis of collagen VI reporter mice revealed collagen VI expression by marginal reticular cells and follicular dendritic cells located in PPs but not in other secondary lymphoid organs, thereby indicating the existence of intestine-specific stromal precursors (53). In addition, M cell–inducing stromal cell populations are positive for collagen IV, again supporting the presence of unique stromal cells in mucosa-associated lymphoid organs (19). Interaction between hematopoietic lymphoid tissue inducer cells (LTi) and stromal lymphoid tissue organizer cells (LTo) establishes a microenvironmental milieu that supports the construction of lymphoid structures through integrin- and chemokine-dependent recruitment of lymphocytes (54) (Figure 2). In fact, organogenesis of PPs is widely accepted to be mediated through interaction between LTi (or PP inducer cells [PPi]) and stromal LTo cells [reviewed in (55)]. At embryonic day 16.5 in mice, aggregates of PPi cells are present at the site of VCAM1^+^ LTo cells in gut (55). The LTi in PPs (i.e., PPi cells) are characterized as CD3^−^CD4^+^IL-7Rα^+^CD44^+^CD90^+^c-kit^+^, and they produce lymphotoxin. Importantly, another cell population—which are RET (rearranged during transfection)^+^CD3^−^CD4^−^IL-7Rα^−^CD11c^+^ cells (so-called PP “initiator” cells)—must be present before clustering of and interaction between LTo and PPi cells can occur at the site of PP organogenesis (55–57). RET is an essential tyrosine kinase receptor for generation of the enteric nervous system; deficiency of RET led to an absence of enteric ganglia and subsequent development of Hirschsprung's disease (56, 58). In addition, the RET ligand artemin (also known as enovin or neublastin; a member of the Glial cell-derived neurotrophic factor ligand family) is produced from LTo cells and recruits PP “initiator” to the anlagen protein via the GFRα3–RET receptor complex (56). Importantly, reduction of CD11c^+^ PP “initiator” cells decreases PP numbers in the CD11c–DTR mouse model, indicating the existence of PP “initiator”-dependent and -independent PP. Whether dependency on CD11c^+^ PP “initiator” signaling, or the machinery of PP organogenesis differs according to the anatomic location of PP (e.g., duodenum, ileum) needs to be investigated.

LTo cells express various adhesion molecules (e.g., VCAM-1 and ICAM-1) in response to lymphotoxin and its receptor, both of which are expressed by LTo cells [(54); reviewed in (55)]. PP LTo cells are considered to be heterogeneous populations, according to their VCAM-1 and ICAM-1 expression levels, and various chemokines, including CXCL13 and CCL21, are abundantly released from VCAM-1^high^ and ICAM-1^high^ stromal cells [reviewed by (59, 60)]. In addition, LTo cells in PP release CCL19/21 and CXCL13, whereas those in the mediastinal lymph nodes release CCL7/11 and CXCL1 (61). Furthermore, mice lacking transcription factors; Id2 and Rorc, which are involved in the development of LTi cells, fail to organize PP (55).

Taking these findings together, stromal cells are involved in and are necessary for mucosa-associated lymphoid organogenesis. However, the nature and fate of various organizer cells (e.g., LTo cells) have not yet been elucidated and merit further research. The possible heterogeneity of LTo cells particularly ought to be investigated, given that the local tissue microenvironment (e.g., stromal cells) may influence this characteristic.

## Stromal–Immune Interaction for Stromal Cell–Mediated Peripheral Differentiation and Functional Modulation of the Intestinal Mucosal Immune System

The seminal influence of stromal cells on the immune system occurs not only in mucosa-associated inductive or organized sites (e.g., gut-associated lymphoid tissue) but also at effector or diffused sites (e.g., intestinal lamina propria). Precise investigations of the immune cells in peripheral tissues revealed the direct interaction between stromal and immune cells (Figure 1). In this regard, activated immune cells stimulate fibroblasts to express Acta2; these induced fibroblasts—due to signaling through TGF-β1—subsequently develop into myofibroblasts [reviewed in (62)].

Several cascades of stromal cell-mediated immune regulation have been reported but remain poorly characterized as yet (63). For example, IgA production, which is critically involved in both innate and acquired mucosal protection (6), is regulated through stromal cell–derived retinoic acid and TGF-β1; these two factors directly and indirectly stimulate mucosal B cells and subsequently lead to the development of IgA-producing plasma cells (64). Another direct pathway involves the secretion of APRIL (a proliferation-inducing ligand) and BAFF (B-cell activating factor) from gut stromal cells; these factors then induce T cells and CD40-independent IgA class switching (65). In addition, an indirect pathway has been reported in which stromal cell-derived type I interferon stimulates plasmacytoid dendritic cells to produce APRIL and BAFF and subsequently enhance IgA production (66). Similarly, retinoic acid and GM-CSF from stromal cells condition dendritic cells to enhance the IgA production pathway (64). Intriguingly, comparison of stromal cell gene expression among tissues reveals that the capability to produce retinoic acid differs among tissues and organs (e.g., colon, small intestine, lung, skin, liver, kidney) (67). Among stromal cells, preferential producers of retinoic acid reside in the small intestine and colon, whereas retinoic acid inactivators or stromal cells that express the enzyme Cyp26b1 occur solely in the cutaneous compartment (67). These data indicate the opposite roles of stromal cells in terms of retinoic acid metabolism in the intestinal mucosa compared with skin (67). Indeed, deficiency of Cyp26b1 or its inhibition by treatment with liarozole induces the expression of retinoic acid–dependent molecules (e.g., P2X7, an ATP receptor) in skin-resident mast cells and T cells, leading to their undesired activation and consequently inflammation (67, 68). Furthermore, whereas ectopic increases in retinoic acid in the cutaneous microenvironment induce chronic inflammation through P2X7 signaling (67), steady-state levels of mucosal P2X7 support the regulation of commensal dysbiosis to maintain homeostasis (69). These findings reveal the absolute requirement of stromal cells for maintenance of tissue-specific homeostasis and their highly variable roles which reflect the local and unique microenvironment of the individual tissue.

In addition to homeostatic regulation through the retinoic acid–dependent pathway, colon stromal cells directly influence T cell activity during inflammation through the programmed death ligand 1 (PD-L1)–programmed cell death protein 1 (PD-1) pathway (70). PD-1–mediated T cell inhibition is an important mechanism for preventing cancer (71, 72). In colon, stromal cells are the predominant PD-L1–expressing cells (73), and co-culture of PD-L1–expressing stromal cells and activated CD4^+^ T cells reduced the production of IFNγ by this T cell population (74). In addition, the expression of PD-L1 at inflammatory sites was increased during ulcerative colitis but reduced during Crohn's disease; these findings indicate that stromal cell–associated suppression (or downregulation) of Th1 responses is diminished in Crohn's disease (74). Stromal PD-L1 expression is increased through TLR signaling (TLR1, 2, 4, and 5) (74), suggesting that exposure to commensal bacterial components during inflammation enhances anti-inflammatory properties of stromal cells-mediated by PD-L1. Importantly, bacterial stimulation is required for stromal cells to acquire IgA-inductive properties (66), thus germ-free conditions reduce the capability of stromal cells to produce IgA-enhancing cytokines (e.g., BAFF, a B-cell activating factor belonging to the TNF family) (66). Together, these findings indicate that the unique properties of intestinal stromal cells depend on input from the commensal microbiota.

During recent years, the importance of stromal cells for the maturation of immune cells in peripheral sites has emerged (20). For example, the maturation of mast cells (i.e., acquisition of granules) requires direct intercellular communication with stromal cells (20). The interaction of c-kit, expressed on mast cells, and stem cell factor on stromal cells is essential for mast cell maturation (75), and a deficiency in c-kit results in a lack of mast cells (75). In addition, group III phospholipase A2 from mast cells reportedly stimulates stromal cells (e.g., fibroblasts), which then produce prostaglandin D2 and bind to DP1 receptors on mast cells for their further activation (76). These two pathways collectively induce granule formation in mast cells. The contents of granules are regulated by stromal cells as well. Co-culture of mast cells with gut-derived stromal cells induces chondroitin sulfate and proteases (e.g., mast cell proteases 1 and 2) (67), all of which are involved in the clearance of parasites (77). Those properties are not induced through co-culture with skin-derived mast cells, indicating that the terminal differentiation and peripheral education of mast cells are tightly regulated by tissue- or organ-derived stromal cells. In addition to their role in mast cell development, stromal cells support the maturation of type 2 innate lymphoid cells (78). Single-cell RNA-Seq analysis revealed that the development and maturation of type 2 innate lymphoid cells are regulated by both PDGFR^−^ and PDGFR^+^ stromal cells through IL-33 and as yet unknown mediators, respectively (78). Furthermore, cooperation between these two stromal cell populations is important to the maintenance of type 2 innate lymphoid cells in the periphery.

Overall, it has become apparent that gut stromal cells act on diverse immune cell populations to support their differentiation and function. Additional studies that explore the crucial roles of the stromal–immune cell axis are essential for understanding how the local microenvironment are regulated. This line of the study will lead to the creation of new research and development platforms for the generation of novel preventive and therapeutic drugs and vaccines that target the stromal–immune cell axis to control inflammation, hypersensitivity, and infection.

## Conclusion and Prospectives

Due to their multifaceted abilities (e.g., lymphoid genesis, peripheral education), stromal cells are important for maintaining the integrity of the intestinal mucosal barrier. In contrast, during inflammation, stromal cells exacerbate the pathologic conditions in fibrosis and carcinoma [reviewed in (79, 80)]. It is important to elucidate the “functional transition” of each stromal cell population from physiologic to pathologic states in the intestinal mucosa. In addition to those in the gut mucosa, stromal cells in other mucosal tissues, such as lung, have important and unique characteristics; the underlying functional mechanisms are gradually being uncovered and are reflecting the anatomic and functional individuality of these cells. For example, detailed analyses in lung stromal cell populations are revealing the genetic (e.g., LGR5^+^, LGR6^+^ populations), anatomic (e.g., bronchiolar, alveolar), and functional (e.g., epithelial differentiation) characteristics of each stromal cell subset (81, 82). However, in gut, even though the functional importance of intestinal stromal cells has gradually been uncovered, the stromal sub-populations are not yet clearly defined. In addition, epithelial–mesenchymal and endothelial–mesenchymal transitions are important pathways that reinforce or compensate for stromal cell populations; these cascades complicate efforts to clarify the fate and origin of stromal cells (83, 84). Further analysis to verify the functional modulations of stromal cells in mucosal health and disease states are required.

## Author Contributions

All authors listed have made a substantial, direct and intellectual contribution to the work, and approved it for publication.

### Conflict of Interest Statement

The authors declare that the research was conducted in the absence of any commercial or financial relationships that could be construed as a potential conflict of interest.
